# Complete Mitochondrial Genome Sequence of *Lichtheimia ramosa* (syn. *Lichtheimia hongkongensis*)

**DOI:** 10.1128/genomeA.00644-14

**Published:** 2014-07-03

**Authors:** Shui-Yee Leung, Yi Huang, Susanna K. P. Lau, Patrick C. Y. Woo

**Affiliations:** a Department of Microbiology, The University of Hong Kong, Hong Kong; b State Key Laboratory of Emerging Infectious Diseases, The University of Hong Kong, Hong Kong; c Research Centre of Infection and Immunology, The University of Hong Kong, Hong Kong; d Carol Yu Centre of Infection, The University of Hong Kong, Hong Kong

## Abstract

We report the complete mitochondrial genome sequence of *Lichtheimia ramosa* (syn. *Lichtheimia hongkongensis*), the first complete mitochondrial DNA sequence of the genus *Lichtheimia*. This 31.8-kb mitochondrial genome encodes 11 subunits of respiratory chain complexes, 3 ATP synthase subunits, 25 tRNAs, and small and large rRNAs, with the gene order *atp9-cox2-atp6-cox3-cox1-nad2-nad3-cob-nad1-nad6-nad5-nad4l-nad4-atp8.*

## GENOME ANNOUNCEMENT

Mucormycosis is an opportunistic infection caused by molds of the order *Mucorales*. The most common species of *Mucorales* associated with mucormycosis belong to the genera *Rhizopus*, *Rhizomucor*, *Mucor*, and *Absidia*. Among the *Absidia* species, the most important species associated with mucormycosis is *Absidia corymbifera*. According to physiological, phylogenetic, and morphological data, it was proposed that three *Absidia* species, *A. corymbifera*, *A. blakesleeana*, and *A. hyalospora*, should be reclassified as a separate family, *Lichtheimiaceae* fam. nov., and the three species renamed *Lichtheimia corymbifera*, *Lichtheimia blakesleeana*, and *Lichtheimia hyalospora* (1, 2). *L. blakesleeana* was subsequently reduced to a synonym of *L. hyalospora* (3). Recently, we and others described a novel *Lichtheimia* species, *Lichtheimia ramosa* (syn. *Lichtheimia hongkongensis*), from patients with mucormycosis (4, 5). We also found that a significant number of reported *L. corymbifera* infections were actually caused by *L. ramosa* (6). Among the species of *Mucorales*, the only complete mitochondrial genome sequenced is that of *Rhizopus oryzae* (7). In this article, we report the complete mitochondrial genome sequence of *L. ramosa* HKU22.

Mitochondrial DNA (mtDNA) of *L. ramosa* was purified using the mitochondrial DNA isolation kit, according to the manufacturer’s instructions (PromoKine), and sequenced using random clone libraries, with the nebulized mitochondrial DNA cloned into a modified pBlueScript KS+ vector. Both DNA strands were sequenced by the dideoxy chain-termination method and assembled using CAP3 (http://pbil.univ-lyon1.fr/cap3.php) (8). The putative open reading frames (ORFs) were denoted using Artemis, with the genetic code of mold. Genes were assigned functions through a BLASTp search against fungal mitochondrion-encoded proteins available in the GenBank database. Introns and rRNAs were identified by a BLASTn pairwise comparison of *L. ramosa* mtDNA with other fungal mitochondrial DNAs. The BLASTn results were viewed through ACT, a DNA sequence comparison viewer based on Artemis, and the exon/intron boundaries were adjusted manually. tRNAs were predicted by tRNAscan-SE 1.21 (http://lowelab.ucsc.edu/tRNAscan-SE/) (9). The core structures of the group I introns were inferred by the program RNAweasel (http://megasun.bch.umontreal.ca/RNAweasel/).

The mtDNA of *L. ramosa* is a circular DNA molecule of 31,830 bp. The G+C content is 34.1%. Genes are carried by both DNA strands, with the gene order *atp9-cox2-atp6-cox3-cox1-nad2-nad3-cob-nad1-nad6-nad5-nad4l-nad4-atp8*. Structural genes occupy 57.9% of the genome (39.1% protein-coding exons, 5.8% tRNA genes, and 13.0% rRNA genes), 8.0% is occupied by intergenic spacers, and 1.9% is occupied by introns. The genome contains 11 genes encoding subunits of respiratory chain complexes (cytochrome oxidase subunits I, II, and III [*cox1*, *cox2*, and *cox3*, respectively], apocytochrome b [*cob*], and the reduced nicotinamide adenine dinucleotide ubiquinone oxireductase subunits [*nad1*, *nad2*, *nad3*, *nad4*, *nad4l*, *nad5*, and *nad6*]) and three genes encoding ATP synthase subunits (*atp6*, *atp8*, and *atp9*). It also encodes 25 tRNAs and the 23S and 16S rRNAs of the large and small subunits of the ribosome (*rnl* and *rns*, respectively). There is one 120-bp ORF, with an unknown function, between *nad4* and *atp8*. The *cox1* and *cox2* genes contain one group I intron each. Phylogenetically, the mtDNA of *L. ramosa* is most closely related to that of *R. oryzae*.

### Nucleotide sequence accession number.

The mtDNA sequence is deposited in GenBank with accession no. KJ561171.
